# An Evidence-Based Review of Related Metabolites and Metabolic Network Research on Cerebral Ischemia

**DOI:** 10.1155/2016/9162074

**Published:** 2016-05-05

**Authors:** Mengting Liu, Liying Tang, Xin Liu, Jing Fang, Hao Zhan, Hongwei Wu, Hongjun Yang

**Affiliations:** Institute of Chinese Materia Medica, China Academy of Chinese Medical Sciences, Beijing 100700, China

## Abstract

In recent years, metabolomics analyses have been widely applied to cerebral ischemia research. This paper introduces the latest proceedings of metabolomics research on cerebral ischemia. The main techniques, models, animals, and biomarkers of cerebral ischemia will be discussed. With analysis help from the MBRole website and the KEGG database, the altered metabolites in rat cerebral ischemia were used for metabolic pathway enrichment analyses. Our results identify the main metabolic pathways that are related to cerebral ischemia and further construct a metabolic network. These results will provide useful information for elucidating the pathogenesis of cerebral ischemia, as well as the discovery of cerebral ischemia biomarkers.

## 1. Introduction

Cerebral ischemia is caused by insufficient blood and oxygen delivery to the brain, which manifests as cerebral death or partial necrosis of the brain. According to the World Health Organization (WHO), ischemia causes 5 million deaths and 5 million cases of irrecoverable disability globally each year (http://www.who.int/en/). Cerebral ischemia is difficult to cure and has a high relapse rate. The specific cause of ischemia is quite complex and the mechanism of pathogenesis remains unclear. Recently, the rapid development of systems biology in areas like genomics, transcriptomics, and proteomics has brought cerebral ischemia research to a new level.

Metabolomics, also called metabonomics, is based on qualitative and quantitative analyses of the end products in specific organisms or cells [1]. In 1970, E. C. Horning and M. G. Horning began to study metabolic profiles of metabolites in humans [2]. In 1982, van der Greef analyzed urine samples by gas chromatography-mass spectrometry (GC-MS) for the first time. This was followed by Nicholson's research that applied nuclear magnetic resonance (NMR) to analyze the metabolic profiles of plasma and urine samples [3–5]. Metabolomics research rapidly progressed during the mid-1990s, when Fiehn and Nicholson defined the concepts of metabolomics and metabonomics, respectively [6, 7]. Acting as a bridge between genotypes and phenotypes, metabolomics can determine comprehensive changes that happen in diseases by analyzing big data pools. Metabolomics studies can clarify specific mechanisms from a systematic perspective by revealing metabolic networks and biomarker groups. When compared to isolated single pathways or single biomarkers, the systemic data are more beneficial for elucidating the pathogenesis of complex diseases like cerebral ischemia [8].

Thus far, the pathogenesis of cerebral ischemia has been linked to energy metabolism, excitatory amino acid toxicity, reactive oxygen species (ROS), and inflammatory responses. These processes involve many kinds of metabolites, whose qualitative and quantitative expression is the focus of metabolomics. This paper introduces the analytical techniques and models used in metabolomics research on cerebral ischemia. Then, the biomarker metabolites in rat cerebral ischemia are summarized. Additionally, based on pathway enrichment analyses, we have successfully determined related metabolic pathways and constructed a metabolic network for rat cerebral ischemia. These novel analyses provide powerful references that clarify cerebral ischemia pathogenesis and reveal related biomarkers.

## 2. Techniques in Metabolomics Research

### 2.1. NMR

NMR is one of the most common techniques used in metabolomics research and has been used since the 1970s [9]. Compared to MS, NMR is a nondestructive test. When samples are difficult to obtain, like cerebrospinal fluid (CFS), digestive fluid, or seminal fluid, NMR is advantageous because it is reproducible, safe, and efficient with the samples. In addition, 1H-NMR can provide robust information on metabolites, and it is advantageous in determining unknown compound structures. However, because NMR is not as sensitive as MS, it is unable to detect molecules at low concentrations [10]. Presently, scientists have successfully applied NMR to construct metabolite profiles from rat tissues, plasma, and human body fluids of cerebral ischemia. Creation of these profiles has promoted research on related pathogenesis and on development of anticerebral ischemia drugs. Importantly, NMR is a powerful tool in the fields of drug toxicity prediction, disease diagnosis, and aging research [1, 11–13].

### 2.2. Chromatography-Coupled MS

GC-MS was the first technique applied to metabolomics research [5]. To use GC-MS for a metabolomics assay, the derivatization step is essential to process biofluid samples like blood and urine [14]. Since commercial structure databases are available for reference, GC-MS is highly advantageous in metabolite identification. In contrast to GC-MS, high-performance liquid chromatography-MS (HPLC-MS) and ultra-performance liquid chromatography-MS (UPLC-MS) techniques do not need the derivatization step. Because they can detect plenary compounds, HPLC-MS and UPLC-MS have become the key techniques used in untargeted and targeted metabolomics [15–17]. Additionally, UPLC use reduces the chromatography running time, making high-throughput analyses achievable [15, 16]. However, techniques for LC-MS are underdeveloped, and there are not comprehensive and unified MS databases for endogenous small molecules. So experience-based reasoning and alignment with standard data are needed to identify the structures of compounds. In addition, the capillary electrophoresis-MS (CE-MS) technique has a high peak capacity and better sensitivity, so it can also be successfully applied [10]. In current cerebral ischemia metabolomics research, LC-MS is the dominant approach used for analyzing plasma, brain tissue, and CFS samples.

## 3. Animals and Models in Cerebral Ischemia Metabolomics Research

Commonly used animals for cerebral ischemia metabolomics research include rats, mice, gerbils, rabbits, dogs, cats, monkeys, and pigs (Table 1). Among these, the rat is most frequently used. Since research on other animals is relatively rare, we chose to summarize biomarkers and conduct functional enrichment analyses from rat cerebral ischemia data. For models, middle cerebral artery occlusion (MCAO) is the most canonical and most common [18]. In the original MCAO model, the exact locations of round tips could not be directly observed. Also, because the round tips cause unexpected reactions that may increase noise signals, MCAO has to be continuously modified. In fact, Shmonin and his colleagues have developed 5 modified MCAO models. Based on the infarct areas and data stability, they selected stable models of permanent cerebral ischemia [19].

## 4. Biomarkers in Rat Cerebral Ischemia

Based on important articles published from 1992 to present, we summarized 120 significantly changed metabolites in cerebral ischemia. All metabolites were presented at supplementary table in Supplementary Material available online at http://dx.doi.org/10.1155/2016/9162074 with KEGG ID and the related detection information. They have been divided into the following 5 categories: amino acids, nucleic acids, neurotransmitters, lipids, and others (mainly organic acids). Samples include plasma, serum, CSF, cortex, hippocampus, striatum, thalamus, midbrain, white matter, pineal body, and olfactory bulb. Of these tissues, plasma, serum, CSF, cortex, hippocampus, and striatum are relatively well studied, while the remaining samples are less studied.

### 4.1. Amino Acids

Table 2 lists 25 amino acids that change in cerebral ischemia. These were measured from plasma, serum, cortex, hippocampus, striatum, thalamus, and midbrain rat tissues. It has been reported that excitatory amino acids (EAA, including glutamic acid and aspartate) and glycine in brain tissues increased 1 hour after reperfusion following ischemia [20, 21]. Wang et al. observed increased levels of glutamic acid in serum and CSF at 24 hours after ischemia/reperfusion, while other amino acids like alanine dynamically decreased and then increased. Glycine and serine levels in CSF continued to decrease in the 6 hours after ischemia [22]. However, not all experiments indicated that excitatory amino acids increased after ischemia/reperfusion. Wang et al. reported that the level of aspartate remained stable at 12 hours after reperfusion, although glutamic acid increased. Other amino acids like leucine, isoleucine, valine, and glutamine were decreased in rat serum and brain extracts [21].

### 4.2. Nucleic Acids

20 of the 120 cerebral ischemia biomarkers are nucleic acids (Table 3). Irie et al. used LC-MS and matrix-assisted laser desorption/ionization-MS (MALDI-MS) techniques to detect 20 nucleic acids in rat cortex, hippocampus, and striatum after reperfusion and compared the results to a normal hemisphere. In cortex and striatum, most nucleic acid levels changed significantly, while levels in the hippocampus remained unchanged. Most nucleic acids, except AMP, constantly decreased during the long time period after reperfusion [23]. Two theories have been proposed to explain these decreases. First, during the early stage of ischemia, the robust synthesis process of excitatory amino acids quickly depletes the nucleic acid pool [24]. Second, the activity of ribose 5-phosphate dehydrogenase, which participates in the pentose phosphate pathway, decreases in ischemia, reducing the total nucleic acid level [25]. Increased AMP levels induce phosphorylation of AMP-activated protein kinase (AMPK), a kinase activated by AMP, further activating phosphofructokinase-2 (PFK-2). In cells that lack oxygen, the PFK-2 activation induces a new round of damage [26].

### 4.3. Neurotransmitters

16 species of neurotransmitter biomarkers in rat cerebral ischemia have been detected in all areas of the brain (Table 4). Neurotransmitters can be divided into two groups: amino acid neurotransmitters and monoamine neurotransmitters. In the amino acid group, glutamate and aspartate are important excitatory neurotransmitters in brain CNS, while GABA and glycine are major inhibitory neurotransmitters. Taurine and serine can inhibit glutamate and GABA receptors. In previous studies, the observed results from amino acid neurotransmitters are not consistent. For example, taurine has been observed to decrease in ischemic tissues like cortex, hippocampus, and whole brain tissue; yet another research has found increased taurine levels in the hippocampus after ischemia [27–29].

In the monoamine group, the testing area and time after ischemia influence the results. In extracellular fluids, the levels of dopamine (DA), norepinephrine (NE), and serotonin (5-HT) increase after ischemia but quickly decrease after reperfusion. DA was increased at 30 minutes after ischemia in rat striatum [30]. DA and 5-HT release were significantly increased at 10 minutes after ischemia in rat nucleus accumbens [31]. However, the DA release was decreased in the nucleus accumbens and cortex of rat bilateral vertebral arteries [32]. Monoamines like DA, NE, and 5-HT do not share the same behavior changes in different cerebral areas, but they are related to free radical production, excitatory cellular toxicity, and cell death [33].

### 4.4. Lipids

Lipid metabolites for cerebral ischemia are very important and getting more attention. MALDI-MS imaging was used to visualize the spatial distribution and concentrations of sulfatide (d18:1-C24h:0), phosphatidylcholine (PCs), and LysoPCs within brain slices of MCAO rats [34, 35]. In clinical metabolomics research, the following six free lipids in CSF were significantly increased: myristic acid, docosahexaenoic acid (DHA), arachidonic acid, linoleic acid, palmitic acid, and oleic acid. Of these, arachidonic acid levels changed most significantly. These results indicated that cerebral ischemia was related to the metabolism of arachidonic acid and DHA, as well as phospholipase activation [36]. It was also reported that, in the first few minutes after ischemia, monounsaturated fatty acids (MUFAs) began to accumulate and continued to increase over hours and days. Furthermore, there was a particularly high increase of MUFAs in the CA1 area of the hippocampus [37–43]. A similar result of increasing abundance of LPC 16:0, LPC 18:0, LPC 18:1, PC 34:0, PC 36:1, and PC 40:6 was stated by MALDI-MS profiling research [35]. Similar to MUFAs, some polyunsaturated fatty acids (PUFAs) like leukotriene C4 and prostaglandin E2 have a fast increase in early periods of ischemia/reperfusion [44]. In addition to mediating multiple important processes in cerebral ischemia, lipids are also involved in the development of Alzheimer's disease (AD), Parkinson's disease (PD), and Niemann-Pick disease [45, 46]. Currently, lipidomics and sterolomics have been used in AD research, independent of metabolomics [47]. Lipidomics is a powerful technique that can also be applied in cerebral ischemia research [48, 49].

### 4.5. Other Metabolites

52 of the 120 metabolites altered in cerebral ischemia are basic metabolites from many important basal metabolic pathways (Table 5). Most of these basic metabolites are organic acids. Some examples are succinate, citrate, malate,* cis*-aconitic acid, and malonic acid, all of which are TCA pathway intermediates. Wang et al. detected citrate and malonic acid accumulation in serum at 0.5 and 3 hours after ischemia, while succinate levels decreased at 24 hours after ischemia [50]. Similar to Wang et al., Irie et al. detected citrate, malate, and* cis*-aconitic acid accumulation in striatum at 3 hours after ischemia [23]. A satisfying explanation for this is that some TCA-related enzymes like aconitase and oxoglutarate dehydrogenase are inhibited, causing cycle suppression and subsequent intermediate accumulation (except succinate) [47]. As for lactate and pyruvate, they are significantly increased after ischemia, indicating that local cerebral ischemia enhances anaerobic metabolism [22, 27, 29, 39, 51–53].

Many basic metabolites, such as UDP, CDP-choline, glucose-6-phosphate (G6p), and UDP-Glucose, participate in biomembrane synthesis. After ischemia, these levels are reduced in cortex and in human acute lymphoblastic leukemia Jurkat cells, which is likely related to the cell membrane damage caused by ischemia [20, 34].

### 4.6. Summary

In early studies of cerebral ischemia, researchers usually prepared whole brain tissue homogenates. Results from these studies indicated the average level of metabolites in the whole brain. Unfortunately, the expression of individual metabolites in different cerebral areas is distinct [52, 54]. For instance, Macrì et al. found that alanine levels are reduced in the hippocampus and yet remain stable in the cortex [55]. Used as an index for ischemia evaluation, the ratio of choline to creatine changes differently in males and females and also in different cerebral areas [56–58]. Therefore, in cerebral ischemia metabolomics research, the influence of both spatial and temporal issues should be fully considered in biomarker discovery. Samples should be restricted to specific cerebral areas to ensure the accuracy of identified biomarkers. With the help of MALDI-MS imaging, we can acquire different metabolite concentrations from each area by spatial localized scans of brain tissue slices [34, 59].

## 5. Functional Enrichment Analyses of Altered Metabolites in Cerebral Ischemia

**Figure 1:**
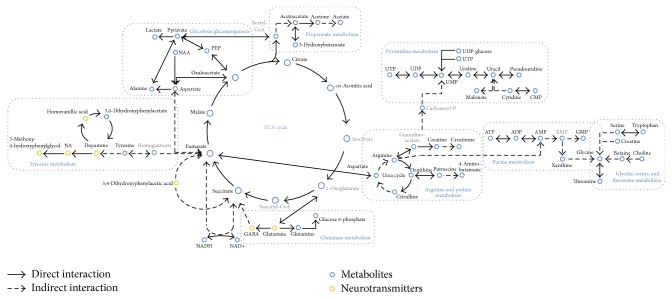
Metabolic network of changed metabolites in cerebral ischemia. Bold type: metabolites already tested in rat. Gray type: metabolites not yet tested in rat.

We used the Metabolite Biological Role (MBRole) website to analyze the enrichment of pathways of the 120 altered metabolites in cerebral ischemia [60]. Then, we set the global metabolites of rats from the KEGG database as background and identified 25 pathways related to cerebral ischemia when the* p* value was set at 0.01. These pathways contained 80 of the 120 metabolites, and isomers were counted as two metabolites. The rest of 13 metabolites lacked pathway annotation in the KEGG, and 27 metabolites were not recorded in the KEGG (supplementary table).  Table 6 lists the top 10 pathways ranked by adjusted* p* values. These data indicate that the biomarkers of rat cerebral ischemia are mainly related to pathways like the TCA cycle, pyrimidine metabolism, tyrosine metabolism, oxidative phosphorylation, and neuroactive ligand-receptor interaction. Based on the pathway enrichment analyses and related information in the KEGG database, we constructed a metabolic network of rat cerebral ischemia (Figure 1). This metabolic network centers on the TCA cycle, surrounded by tyrosine metabolism, alanine, aspartate and glutamate metabolism, pyrimidine metabolism, and pyridine metabolism. It contains 53 of the 120 metabolites. The network only represents the relationships among altered metabolites. Due to variance in many factors like exact area of sampling, time of sampling, and the models applied, the dynamic behaviors of each metabolite were not considered in the construction of this network.

In addition, three important pathways including Parkinson's disease, oxidative phosphorylation, and neuroactive ligand-receptor interaction had either relatively distant relationships with others or lacked supportive literature. Consequently, they were not included in the basic network.

Parkinson's disease is a progressive neurodegenerative movement disorder that is mainly caused by nigrostriatal dopaminergic neuron death [61]. Currently, there are 13 small molecules reported to participate in the pathogenesis of PD according to KEGG (ATP, ADP, orthophosphate, diphosphate, AMP, hydrogen peroxide, L-tyrosine, adenosine, 3,4-dihydroxy-L-phenylalanine, 3′,5′-cyclic AMP, superoxide anion, dopamine, 1-methyl-4-phenyl-1,2,3,6-tetrahydropyridine, rotenone, and 1-methyl-4-phenylpyridinium). Five cerebral ischemia biomarkers are involved in the PD pathway: ATP, AMP, ADP, tyrosine, and dopamine. The results of pathway enrichment analyses indicate a relationship between cerebral ischemia and PD pathogenesis.

Oxidative phosphorylation is an essential energy metabolism pathway occurring in mitochondria. This pathway contains 16 metabolites in total. Fumarate, succinate, ADP, ATP, and NAD+ were enriched and are considered to be the biomarkers most related to energy metabolism. All of these are members of the electron transfer chain. NADH is oxidized to NAD+ by dehydrogenase and simultaneously releases an electron. The transformation of succinate to fumarate is part of the electron transfer chain and the TCA cycle. Previous research reported that the intracellular ATP/ADP ratio was very high in cerebral ischemia [62, 63]. The ratios of ATP to ADP and NADH to NAD+ may be cerebral ischemia biomarkers.

Neuroactive ligand-receptor interaction is a signal transduction molecular pathway that plays a key role in neurotransmitter release. 14 of the altered metabolites in cerebral ischemia are components of this pathway: dopamine, serotonin, norepinephrine, ATP, ADP, UTP, UDP, GABA, glutamate, N-acetylaspartylglutamate, taurine, glycine, homocysteine, and alanine. Their involvement proves that altered mechanisms of neuroactive agents are associated with this pathway.

## 6. Conclusion and Discussion

In metabolomics research, the identification of metabolites is truly an arduous task. The existence of isomers, as well as overlapping peaks in NMR profiles, makes it difficult to identify specific compounds based on individual MS or NMR information [97]. For the MS technique, the present strategy is to upgrade the working resolution to improve compound composition accuracy and then verify the compounds by secondary-MS of the pyrolysis fragments. As for NMR, common strategies applied to identify complicated compounds include increasing the magnetic field intensity and use of multiple-nuclear NMR (1H, 13C, 15N, etc.) or multiple-dimension NMR editing (2D-COSY, NOESY, TOCSY, HSQC, etc.) [98–100]. The list of 120 currently known metabolites summarized in this paper provides an informative reference for quickly identifying cerebral ischemia metabolites.

Our explanation for the pathways found by functional enrichment analyses in cerebral ischemia will provide useful information for elucidating the pathogenesis of cerebral ischemia. The metabolic network we constructed will be useful in selecting molecular targets and clarifying the molecular mechanisms of cerebral ischemia. It should be noted that, due to the technical level of analytical instruments, current metabolomics technologies are not really global and not every metabolite can be accurately measured. In every piece of research, analytical instrument types, sample types, sampling time, and researcher skills all may influence the results of metabolomics study and the obtained biomarkers. So the enrichment analysis based on the metabolites from the literature will certainly have biases. However, up to now there are no papers summarizing the metabolic changes in cerebral ischemia nor any related databases. Our review will provide useful information for future research in this field.

## Supplementary Material

The supplementary table contains all the 120 changed metabolites in rat cerebral ischemia with KEGG compound identifiers, the detecting regions and the references.

## Figures and Tables

**Table 1:** Contrast of different methods/models and animals in cerebral ischemia.

Model		Animal	Feature	Reference
Global cerebral ischemia	Two-vessel occlusion (2VO)	Rat	High success rate, obviously damaged after ischemia/reperfusion; inducing whole-body hypotension in model preparation; influencing blood supply of other organs; it cannot be prepared in awake state, so neurobehavior assessment is infeasible	[64, 65]
	Three-vessel occlusion (3VO)	Rat	Rapidly and effectively triggering ischemia; quickly recovering after reperfusion; suitable for acute whole-brained ischemia case, severe operation wound	[66, 67]
	Four-vessel occlusion (4VO)	Rat, rabbit	Suitable for subacute case; it can operate in both anesthetized and awake states; reperfusion is feasible; high mortality rate of animals	[68]

Focal cerebral ischemia	Craniotomy method	Rat, mouse, cat, dog, pig	Accurate and reliable, consistent experimental conditions, high success rate, severe surgical damage; it cannot apply reperfusion, intracranial pressure increase in surgery, damage of blood-brain barrier, change of brain temperature	[69–73]
	Thromboembolic model	Rat, mouse	Imitating in situ cerebral ischemia; it can evaluate the efficacy of thrombolytic agents, three types including microemboli suspension, single embolus, and multiemboli model	[69, 74–76]
	Nonclot embolic model	Rat, mouse, monkey	Using artificial materials to replace natural clot to avoid self-thrombolysis; the volume of embolus is adjustable and able to totally block the target artery, reduce the influence of uncontrollable reperfusion, and precisely control the time point of ischemia/reperfusion and may cause inflammatory response	[77–79]
	Intraluminal suture model	Rat, mouse	Well-reproducible, precise site of damage, precisely controllable time of ischemia; the operation of filament insertion into cranium cannot be directly observed and may cause hemorrhage and/or vasospasm	[80–82]
	Chemical induction model	Rat, mouse	Chemicals stimulate the vessels and induce vasoconstriction or directly produce clots	[83–86]

**Table 2:** Amino acids metabolites in cerebral ischemia.

Metabolites	Plasma	Serum	CSF	Cortex	Hippocampus	Striatum	Thalamus	Midbrain	Whole brain tissue	Reference
Tyrosine		√		√		√				[20, 22, 23]
Serine		√	√							[50]
Dopamine				√	√	√	√	√		[20, 22, 23, 50, 51, 55]
Alanine	√	√	√	√	√					[20, 22, 23, 27]
Citrulline				√		√				[23]
Methionine		√								[20]
*γ*-Aminobutyric acid (GABA)			√	√	√					[23, 27, 28, 55]
Threonine		√								[50]
Glutamate	√			√	√	√				[21, 23, 27, 52, 55]
Valine	√		√	√						[21, 50]
Tryptophan		√		√	√					[20, 23]
Serotonin				√	√	√	√	√		[51]
Glycine		√	√						√	[22, 28, 50]
Phenylalanine	√			√		√				[21, 23]
Glutamine				√		√			√	[23, 27, 28, 55, 87]
Glutamic acid		√	√						√	[20–22, 50]
Histidine				√		√			√	[23]
Aspartate		√		√		√			√	[20, 23, 27, 52]
Isoleucine	√		√						√	[21]
Leucine	√		√							[21]
Nicotinuric acid				√		√				[27]
Homocysteine		√								[20]
Lysine			√							[21]
Ornithine		√								[50]
Arginine				√		√				[50]

**Table 3:** Nucleic acids metabolites in cerebral ischemia.

Metabolites	Cortex	Striatum	Whole brain tissue	Reference
UMP	√	√		[23]
UDP	√	√	√	[23, 27]
UDP-glucose	√	√		[23]
UTP			√	[27]
Uridine	√	√		[23]
Uracil	√	√	√	[23, 27]
Guanosine	√	√		[23]
GMP	√	√		[23]
Cytidine	√	√		[23]
CDP-choline	√	√		[23]
CMP	√	√		[23]
ATP	√	√	√	[23]
AMP	√	√		[23]
ADP	√	√		[23]
Ribose 5-phosphate	√	√		[23]
Neu5Ac	√	√		[23]
Xanthine	√	√	√	[23, 27]
Pseudouridine	√	√		[23]
PE	√	√		[23]
PEP	√	√		[23]

UMP: uridine monophosphate; UDP: uridine diphosphate; UTP: uridine-5′-triphosphate; GMP: guanosine 5′-phosphate; CDP-choline: cytidine 5′-diphosphocholine; CMP: cytidine-5′-monophosphate; ATP: adenosine 5′-triphosphate; AMP: adenosine 5′-monophosphate; ADP: adenosine 5′-diphosphate; Neu5Ac: N-acetylneuraminate; PE: phosphoethanolamine; PEP: phosphoenolpyruvate.

**Table 4:** Neurotransmitter metabolites in cerebral ischemia.

Metabolites	Plasma	Serum	CSF	Cortex	Hippocampus	Striatum	Thalamus + midbrain	White matter	Whole brain tissue	Reference
Dopamine (DA)				√	√	√	√			[30–32, 51]
Norepinephrine (NE)				√	√	√	√			[51]
Serotonin (5-HT)				√	√	√	√			[31, 32, 51]
3-Methoxy-4-hydroxyphenylglycol (MHPG)				√	√	√	√			[51]
3,4-Dihydroxyphenylacetic acid (DOPAC)				√	√	√	√			[51]
Homovanillic acid (HVA)				√	√	√	√			[51]
5-Hydroxyindoleacetic acid (5-HIAA)				√	√	√	√			[51]
GABA			√	√	√					[23, 27, 28, 55]
Glutamic acid		√	√						√	[20, 22, 28, 50]
Glycine		√	√						√	[22, 28, 50]
Glutamate	√			√	√	√				[21, 23, 27, 52, 55]
Aspartate										[20, 23, 27, 28]
Taurine				√		√			√	[27–29]
Serine		√	√							[22, 50]
Choline				√	√			√	√	[27, 52, 54, 55, 88, 89]
*γ*-Hydroxybutyrate						√				[53]

**Table 5:** Other metabolites in cerebral ischemia.

Metabolites	Plasma	Serum	CSF	Cortex	Hippocampus	Striatum	White matter	Thalamus	Midbrain	Whole brain tissue	Pineal body, olfactory bulb	Reference
Choline phosphate										√		[23]
Malate				√		√				√		[23, 27]
Citrate			√	√		√						[23, 50]
Succinate		√								√		[27]
Creatinine			√									[21, 22, 29]
2-Hydroxybutyric acid			√									[22, 53]
Creatine			√	√	√	√						[23, 27, 55, 89]
Glutamate	√			√	√	√				√		[21, 23, 27, 52, 54, 55]
*cis*-Aconitic acid		√		√		√						[23]
Malonate		√										[50]
Alpha-D-glucose										√		[22, 27]
Carnosine	√											[21]
Nicotinamide adenine dinucleotide (NAD+)				√		√						[23]
N^G^-nitro-L-arginine methyl nester (L-NAME)						√						[90]
N-Acetylaspartate (NAA)	√		√	√	√	√	√			√		[23, 52, 54, 55, 88, 89]
N-Acetylaspartylglutamate (NAAG)				√		√				√		[23, 91]
6-Deoxy-6-[18F]fluoro-L-ascorbic acid											√	[92]
3-Methoxy-4-hydroxyphenylglycol (HMPG)				√	√	√		√	√			[51]
3-Hydroxypropyl mercapturic acid				√	√	√		√	√			[23, 93]
Glycerophosphoric acid				√		√						[23]
Glutathione (GSH)				√		√						[23, 24]
Disulfide (GSSG)				√		√						[23]
Myo-inositol			√	√	√	√						[27, 54, 55]
Betaine		√										[50]
Pyruvate		√	√									[22, 50]
Lactate		√	√	√	√	√		√	√	√		[50, 52, 54, 55, 88]
3,4-Dihydroxyphenylacetate (DOPAC)				√	√	√		√	√			[51]
Homovanillic acid (HVA)				√	√	√		√	√			[51]
Nicotinuric acid										√		[27]
Fumarate										√		[27]
Glucose 6-phosphate				√		√						[23]
Formate										√		[27]
Acetate			√							√		[27]
Ascorbate				√		√				√		[23]
Taurine				√		√				√		[23, 27, 28]
Alpha-2-Ketoisovaleric acid			√									[22]
3-Hydroxybutanoic acid			√									[22]
3-Hydroxyisovalerate			√									[22]
Acetone			√									[22]
Acetic acid			√									[22, 50]
Oxaloacetate			√									[22]
Dimethylamine			√									[22]
Glycerol			√									[22]
D-Fructose			√									[22]
Aminoguanidine										√		[94]
Polyamines				√	√					√		[95]
Putrescine				√	√					√		[34, 96]
Spermidine				√	√					√		[34, 59]
Spermine				√	√					√		[34, 59]

**Table 6:** Top 10 relational pathway list (*p* < 0.01).

Label	*p* value	Adjusted *p* value	In background	In set	In set/in background%
Metabolic pathways	5.51*E* − 13	4.24*E* − 11	1455	61	4.2
Citrate cycle (TCA cycle)	4.39*E* − 09	1.69*E* − 07	20	8	40.0
Pyrimidine metabolism	4.45*E* − 07	1.14*E* − 05	59	10	16.9
Tyrosine metabolism	4.99*E* − 06	8.56*E* − 05	76	10	13.2
Parkinson's disease	5.56*E* − 06	8.56*E* − 05	13	5	38.5
Glycine, serine, and threonine metabolism	9.36*E* − 06	1.12*E* − 04	49	8	16.3
Alanine, aspartate, and glutamate metabolism	1.01*E* − 05	1.12*E* − 04	24	6	25.0
Oxidative phosphorylation	1.79*E* − 05	1.64*E* − 04	16	5	31.3
Neuroactive ligand-receptor interaction	1.91*E* − 05	1.64*E* − 04	128	15	11.7
Butanoate metabolism	2.21*E* − 05	1.70*E* − 04	40	7	17.5
