# Supplementary material for: An Evidence-Based Review of Related Metabolites and Metabolic Network Research on Cerebral Ischemia
Source: Oxid Med Cell Longev. 2016 May 5;2016:9162074. doi: 10.1155/2016/9162074 (PMC4871976; doi:10.1155/2016/9162074)
Supplement: Supplementary file 1 — The supplementary table contains all the 120 changed metabolites in rat cerebral ischemia with KEGG compound identifiers, the detecting regions and the references. [file 9162074.f1.pdf]

No.	Metabolites	KEGG ID	Plasma	Serum	CSF	Cortex	Hippocampus	Striatum	Thalamus + Midbrain	White matter	Whole Brain tissue	Pineal body ,Olfactory bulb	Reference
1	2-Hydroxybutyric acid	C05984			√								[45, 65]
2	3,4-dihydroxyphenylacetic acid (DOPAC)	C01161				√	√	√	√				[48]
3	3-Hydroxybutanoic acid	C01089			√								[45]
4	3-Hydroxyisovalerate	C20827			√								[45]
5	3-hydroxypropyl mercapturic acid	No KEGG ID				√	√	√		√			[46, 83]
6	3-methoxy-4-hydroxyphenylglycol(HMPG)	C05583				√	√	√		√			[48]
7	5-hydroxyindoleacetic acid (5-HIAA)	C05635				√	√	√	√				[48]
8	6-Deoxy-6-[18F]Fluoro-L-Ascorbic Acid	No KEGG ID										√	[82]
9	Acetate	C00033			√						√		[45, 47, 50]
10	Acetone	C00207			√								[45]
11	ADP	C00008				√		√					[46]
12	Alanine	C01401	√	√	√	√	√						[43, 45, 46, 50]
13	Alpha-2-Ketoisovaleric acid	C00141			√								[45]
14	alpha-D-Glucose	C00267									√		[45, 50]
15	Aminoguanidine	No KEGG ID									√		[85]
16	AMP	C00020				√		√					[46]
17	Arginine	C02385				√		√					[47]
18	Ascorbate	C00072				√		√			√		[46]
19	Aspartate	C16433$^{\#}$		√		√		√			√		[43, 46, 50, 51,52]
20	ATP	C00002				√		√			√		[46]

21	Betaine	C00719		√								[47]
22	Carnosine	C00386	√									[44]
23	CDP-choline	C00307				√		√				[46]
24	Choline	C00114				√	√			√	√	[49, 50, 52, 62-64]
25	Choline phosphate	C00588									√	[46]
26	cis-Aconitic acid	C00417		√		√		√				[46]
27	Citrate	C00158			√	√		√				[46, 47]
28	Citrulline	C00327				√		√				[46]
29	CMP	C00055				√		√				[46]
30	Creatine	C00300			√	√	√	√				[46, 49, 50, 64]
31	Creatinine	C00791			√							[44, 45, 57]
32	Cytidine	C00475				√		√				[46]
33	D-Fructose	C00095			√							[45]
34	Dimethylamine	C00543			√							[45]
35	Disulfide(GSSG)	C00127				√		√				[46]
36	Dopamine(DA)	C03758				√	√	√	√			[43, 45-49,58-60]
37	Formate	C00058									√	[50]
38	Fumarate	C00122									√	[50]
39	GABA	C00334			√	√	√					[46, 49-51]
40	Glucose 6-phosphate	C00092				√		√				[46]
41	L-Glutamate	C00025	√	√	√	√	√	√			√	[43-47, 49-53]
42	Glutamate	C00302	√	√	√	√	√	√			√	[43-47, 49-53]
43	Glutamine	C00303#				√		√			√	[46, 49-51, 53]
44	Glutathione(GSH)	C00051				√		√				[46, 54]

45	Glycerol	C00116			√							[45]
46	Glycerophosphoricacid	C00093				√		√				[46]
47	Glycine	C00037		√	√						√	[45, 47, 51]
48	GMP	C00144				√		√				[46]
49	Guanosine	C00387				√		√				[46]
50	Histidine	C00768$^{\#}$				√		√			√	[46]
51	Homocysteine	C05330$^{\#}$		√								[43]
52	Homovanillicacid (HVA)	C05582				√	√	√	√			[48]
53	Isoleucine	C16434$^{\#}$	√		√						√	[44]
54	Lactate	C01432$^{\#}$		√	√	√	√	√		√	√	[47, 49, 52, 62, 63]
55	Leucine	C16439$^{\#}$	√		√							[44]
56	leukotriene C4	C02166									√	[84]
57	Lysine	C16440$^{\#}$			√							[44]
58	L-Malate	C00149				√		√			√	[46, 50]
59	Malonate	C00383		√								[47]
60	L-Methionine	C00073		√								[43]
61	Myo-Inositol	C00137			√	√	√	√				[49, 50, 62]
62	N-acetylaspartate（NAA）	C01042	√		√	√	√	√	√		√	[46, 49, 52, 62-64]
63	N-Acetylaspartylglutamate（NAAG）	No KEGG ID				√		√			√	[46, 81]
64	NAD+	C00003				√		√				[46]
65	neu5Ac	C00270				√		√				[46]
66	NG-nitro-L-arginine methyl nester	No KEGG ID						√				[80]

67	Nicotinuric acid	C05380				√		√			[50]
68	Norepinephrine (NE)	C00547				√	√	√	√		[48]
69	Ornithine	C01602#		√							[47]
70	Oxaloacetate	C00036			√						[45]
71	PE	No KEGG ID				√		√			[46]
72	PEP	C00074				√		√			[46]
73	Phenylalanine	C02057#	√			√		√			[44, 46]
74	polyamines	No KEGG ID				√	√			√	[86]
75	prostaglandin E2	C00584								√	[84]
76	pseudouridine	C02067				√		√			[46]
77	putrescine	C00134				√	√			√	[66, 87]
78	Pyruvate	C00022		√	√						[45, 47]
79	Ribose 5-phosphate	C00117				√		√			[46]
80	L-Serine	C00065		√	√						[45, 47]
81	Serotonin(5-HT)	C00780				√	√	√	√		[48, 59, 60]
82	spermidine	C00315				√	√			√	[66, 88]
83	spermine	C00750				√	√			√	[66, 88]
84	Succinate	C00042		√						√	[50]
85	Taurine	C00245				√		√		√	[46, 50, 51, 57]
86	L-Threonine	C00188		√							[47]
87	D-Threonine	C00820		√							[47]
88	L-Tryptophan	C00078		√		√	√				[43, 46]
89	Tryptophan	C00806#		√		√	√				[43, 46]
90	L-Tyrosine	C00082		√		√		√			[43, 45, 46]

91	D-Tyrosine	C06420[#]		√		√	√		[43, 45, 46]
92	UDP	C00015				√	√	√	[46, 50]
93	UDP-glucose	C00029				√	√		[46]
94	UMP	C00105				√	√		[46]
95	Uracil	C00106				√	√	√	[46, 50]
96	Uridine	C00299				√	√		[46]
97	UTP	C00075						√	[50]
98	Valine	C16436[#]	√		√	√			[44, 47]
99	Xanthine	C00385				√	√	√	[46, 50]
100	γ-Hydroxybutyrate	C00989					√		[65]
101	sulfatide (d18:1-C24h:0)	No KEGG ID						√	[66]
102	PC 32:0	No KEGG ID				√	√	√	[67]
103	PC 34:1	No KEGG ID				√	√	√	[67]
104	PC 36:4	No KEGG ID				√	√	√	[67]
105	SM 18:0	No KEGG ID				√	√	√	[67]
106	LPC 16:0	No KEGG ID				√	√	√	[67]
107	LPC 18:1	No KEGG ID				√	√	√	[67]
108	LPC 18:0	No KEGG ID				√	√	√	[67]

109	PC 34:0	No KEGG ID	√	√	√	[67]
110	PC 36:1	No KEGG ID	√	√	√	[67]
111	PC 40:6	No KEGG ID	√	√	√	[67]
112	Ceramide 18:0-H2O	No KEGG ID	√	√	√	[67]
113	PC 16:0/16:0	No KEGG ID	√	√	√	[67]
114	PC 16:0/18:1	No KEGG ID	√	√	√	[67]
115	PC 16:0/18:0	No KEGG ID	√	√	√	[67]
116	PC 16:0/20:4	No KEGG ID	√	√	√	[67]
117	PC 18:0/18:1	No KEGG ID	√	√	√	[67]
118	PC 16:0/22:6	No KEGG ID	√	√	√	[67]
119	PC 18:0/20:4	No KEGG ID	√	√	√	[67]
120	PC 18:0/22:6	No KEGG ID	√	√	√	[67]

C00000#, lacked pathway annotation in KEGG
